# A case of familial hypocalciuric hypercalcemia type 1 due to CASR p.Pro55Leu mutation

**DOI:** 10.1186/s12902-022-01077-5

**Published:** 2022-06-22

**Authors:** Akira Sumida, Katsumi Iizuka, Takehiro Kato, Yanyan Liu, Sodai Kubota, Saki Kubota-Okamoto, Teruaki Sakurai, Toshinori Imaizumi, Yoshihiro Takahashi, Masami Mizuno, Ken Takao, Takuo Hirota, Tetsuya Suwa, Yukio Horikawa, Mayumi Yamamoto, Yusuke Seino, Atsushi Suzuki, Daisuke Yabe

**Affiliations:** 1 Department of Diabetes, Endocrinology and Metabolism/Department of Rheumatology and Clinical Immunology, Gifu University Graduate School of Medicine, Gifu University Graduate School of Medicine, Gifu, 501-1194 Japan; 2 Department of Clinical Nutrition, Fujita Health University, 1-98 Dengakugakubo, Kutsukake-Cho, Toyoake, Aichi 470-1192 Japan; 3 Yutaka Seino Distinguished Center for Diabetes Research, Kansai Electric Power Medical Research Institution, Kobe, Japan; 4 Center for Diabetes, Metabolism, and Endocrinology, Kansai Electric Power Hospital, Osaka, Japan; 5 Health Administration Center, Gifu University, Gifu, Japan; 6 The Department of Endocrinology, Diabetes and Metabolism, Fujita Health University, Toyoake, Japan; 7 Division of Molecular and Metabolic Medicine, Department of Physiology and Cell Biology, Kobe University Graduate School of Medicine, Kobe, Japan; 8 Center for Healthcare Information Technology (C-HIT), Tokai National Higher Education and Research System, Nagoya, Japan

**Keywords:** Familial hypocalciuric hypercalcemia, FHH, Valcium creatinine clearance ratio, CCCR, Calcium-sensing receptor, *CASR*

## Abstract

**Background:**

Familial hypocalciuric hypercalcemia (FHH) is a rare autosomal dominant disease, which requires differential diagnosis from relatively common primary hyperparathyroidism (PHPT) in order to avoid unnecessary surgery.

**Case presentation:**

A 16-year-old female had been followed by the department of psychosomatic medicine at our institution. Throughout the follow-up period, her plasma calcium levels were high, plasma Pi levels were relatively low, and plasma intact PTH was relatively high. She was referred to our department to determine the cause of her hypercalcemia. Her 24 h urinary calcium excretion was as low as 100 mg/day, and calcium creatinine clearance ratio was below 0.01. Moreover, she had a family history of hypercalcemia (proband, her brother, and her father). The genetic testing for her family revealed that she, her brother, and her father were definitively diagnosed with FHH type 1 due to the heterozygous calcium-sensing receptor mutation (NM_00388:4:c.164C > T:p.Pro55Leu).

**Conclusion:**

We experienced a 16-year-old female with FHH, in whom genetic testing identified the heterozygous calcium-sensing receptor mutation (NM_00388:4:c.164C > T:p.Pro55Leu) as pathogenic, permitting a definitive diagnosis of FHH type 1. The genetic testing for calcium sensing receptor is beneficial to distinguish asymptomatic primary hyperparathyroidism from FHH.

## Background

Hypercalcemia is a condition causing disorders in multiple organs, including bone, brain, and heart [1]. The causes of hypercalcemia include hyperparathyroidism, cancer, tuberculosis, immobility, and inappropriate vitamin D supplementation. Among these, primary hyperparathyroidism (PHPT) is a relatively common disease (incidence rate;1 in 1,000) and often requires parathyroid gland resection [2]. Familial hypocalciuric hypercalcemia (FHH) is a rare autosomal dominant disease (incidence rate; 1 in 78,000) that requires, in general, no treatment [2, 3]. Thus, differential diagnosis of FHH from PHPT is critical to avoid unnecessary surgery.

Urinary calcium excretion and calcium creatinine clearance ratio (CCCR) levels are widely used for differential diagnosis of FHH and PHPT; even so, it is sometimes difficult to distinguish the two diseases [2–6]. Approximately 80% of FHH patients demonstrate urinary calcium excretion < 100 mg/day and CCCR < 0.01, while ~ 20% show CCCR 0.01–0.02 [6]. In contrast, PHPT patients usually have CCCR > 0.02, while ~ 20%, who have concomitant vitamin D deficiency show CCCR < 0.01 [6]. It is therefore difficult to distinguish FHH from PHPT based only on CCCR values. Thus, genetic testing becomes critical for the positive diagnosis of FHH.

To date, FHH-related mutations have been identified in genes related to the calcium sensing system that regulates calcium homeostasis [2]. The calcium-sensing receptor, CASR, is encoded by the *CASR* gene and belongs to a G protein-coupled receptor family that regulates parathyroid hormone (PTH) secretion, vitamin D synthesis, and calcium absorption and resorption [2, 5]. G-protein α11 (GNA11) is encoded by the *GNA11* gene and mediates CaSR signaling [2, 5]. Adaptor-related protein complex 2 (AP-2) is a membrane-associated heterotetramer complex and composed of two large chain, medium chain, and a small chain. The adaptor-related protein complex 2 sigma one subunit encoded by the AP2S1 gene binds to CASR and facilitates CaSR internalization [2, 5]. Loss of function mutations in the *CASR*, *GNA11,* or *AP2S1* genes cause FHH type 1 (FHH1), FHH type 2 (FHH2), and FHH type 3 (FHH3), respectively [1, 4]. Approximately 60% of FHH patients have mutations in the *CASR* gene [2].

Here we report a case of FHH1 in which genetic testing identified the heterozygous *CASR* mutation p.Pro55Leu, which permitted the definitive diagnosis of the disease. In this case with no symptom, family history and genetic test for CASR was beneficial for the diagnosis of FHH type1.

## Case presentation

**Fig. 1:**
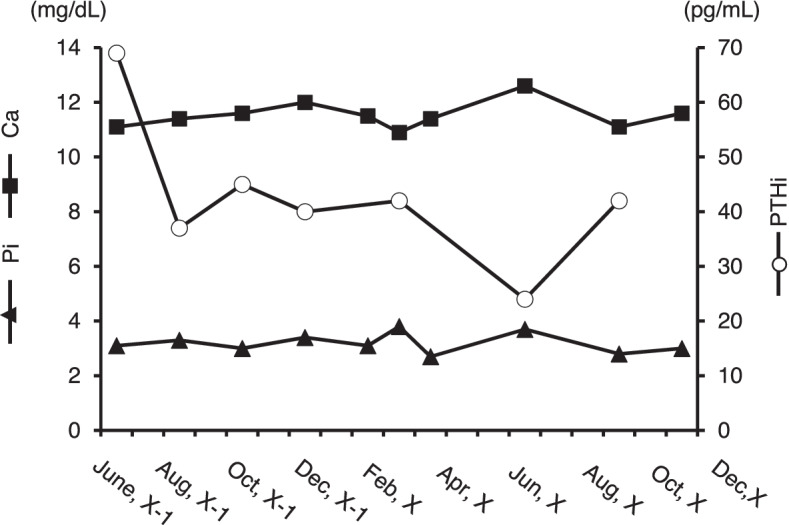
Changes in plasma calcium, inorganic phosphate and intact parathyroid hormone in the current case. Square, plasma calcium; triangle, inorganic phosphate (Pi); circle, intact PTH (PTHi)

A 16-year-old female had been followed by the department of psychosomatic medicine at our institution for borderline personality disorder. Throughout the follow-up period, her plasma calcium levels were high, plasma Pi levels were relatively low, and plasma intact PTH (PTHi) was relatively high (Fig. 1).

Her height and body weight were 154 cm and 53. 5 kg, respectively. Although she was asymptomatic, she was referred to our department to determine the cause of her hypercalcemia on June. Upon referral, her plasma calcium, inorganic phosphate (Pi) and PTHi were 11.5 (normal range; 8.8–10.1) mg/dL, 3.1 (normal range; 2.7–4.6) mg/dL, and 51 (normal range; 10–65) pg/mL, respectively (Table 1 and Fig. 1). Her 1,25-OH vitamin D (1,25-OHD) was elevated (59.7 [normal range; 20.0–60.0]) while 25-OH vitamin D (25-OHD) was low (9 [normal range; < 25]) (Table 1). Thyroid echography and 99mTc-MIBI scintigraphy revealed no abnormalities in the parathyroid glands. Her 24 h urinary calcium excretion was as low as 100 (normal range; 100–300) mg/day, and CCCR was below 0.01 (1^st^ measurement, 0.0053 and 2^nd^ measurement, 0.0059) (normal range; > 0.01) (Table 1). Her 24 h urinary Pi excretion was 0.58 (normal range; 0.5–1.0) g/day, and tubular reabsorption of phosphate was 89.9% (normal range; 80–94) (Table 1). Her TmP/GFR was almost same as plasma Pi levels, which were consistent with her normal renal function (Table 1).

**Table 1:** Laboratory data of this patient

	This patient	Normal ranges
Blood parameters		
Albumin (g/dL)	4.7	4.1–5.1
Creatinine (mg/dL)	0.54	0.46–0.79
Na^+^ (mEq/L)	138	138–145
K^+^ (mEq/L)	3.9	3.6–4.8
Ca^2+^ (mg/dL)	11.5	8.8–10.1
Pi^2−^ (mg/dL)	3.1	2.7–4.6
Mg^2+^ (mg/dL)	2.2	1.8–2.4
Alkaline phosphatase (U/L)	236	106–322
1,25OHD (pg/mL)	59.7	20.0–60.0
25OHD (pg/mL)	9	< 25
PTH intact (pg/mL)	51	10–65
PTH whole (pg/mL)	44.8	8.3–38.7
PTHrP (pmol/L)	< 1.0	≦1.1
TSH (μIU/mL)	0.49	0.54–4.26
FT_4_ (ng/dL)	0.93	0.76–1.65
ACTH (pg/mL)	32.1	7.2–63.3
Cortisol (μg/dL)	15.9	6.24–18.0
Urine parameters		
24 h urinary Ca excretion (g)	0.1	0.1–0.3
24 h urinary Pi excretion (g)	0.58	0.5–1.0
%TRP (%)	89.9	80–94
TmP/GFR (mg/dL)	2.9	2.3–4.3
Calcium creatinine clearance Ratio (CCCR)	0.0059/0.0054	> 0.01

TmP/GFR was estimated by nomogram for derivation of renal threshold phosphate concentration^16^. CCCR were calculated by plasma and 24-h urine samples for calcium and creatinine

*Abbreviations:* *Cre* Creatinine, *1,25OHD* 1,25-hydroxy-vitamin D, *25OHD* 25-hydroxy-vitamin D, *PTH* Parathyroid Hormone, *PTHrP* Parathyroid horimone related Peptide, *TRP* Tubular Reabsorption of Phosphate, *TmP/GFP* Tubular maximum Phosphate reabsorption per GFR, *CCCR* Calcium Creatinine Clearance Ratio.

**Fig. 2:**
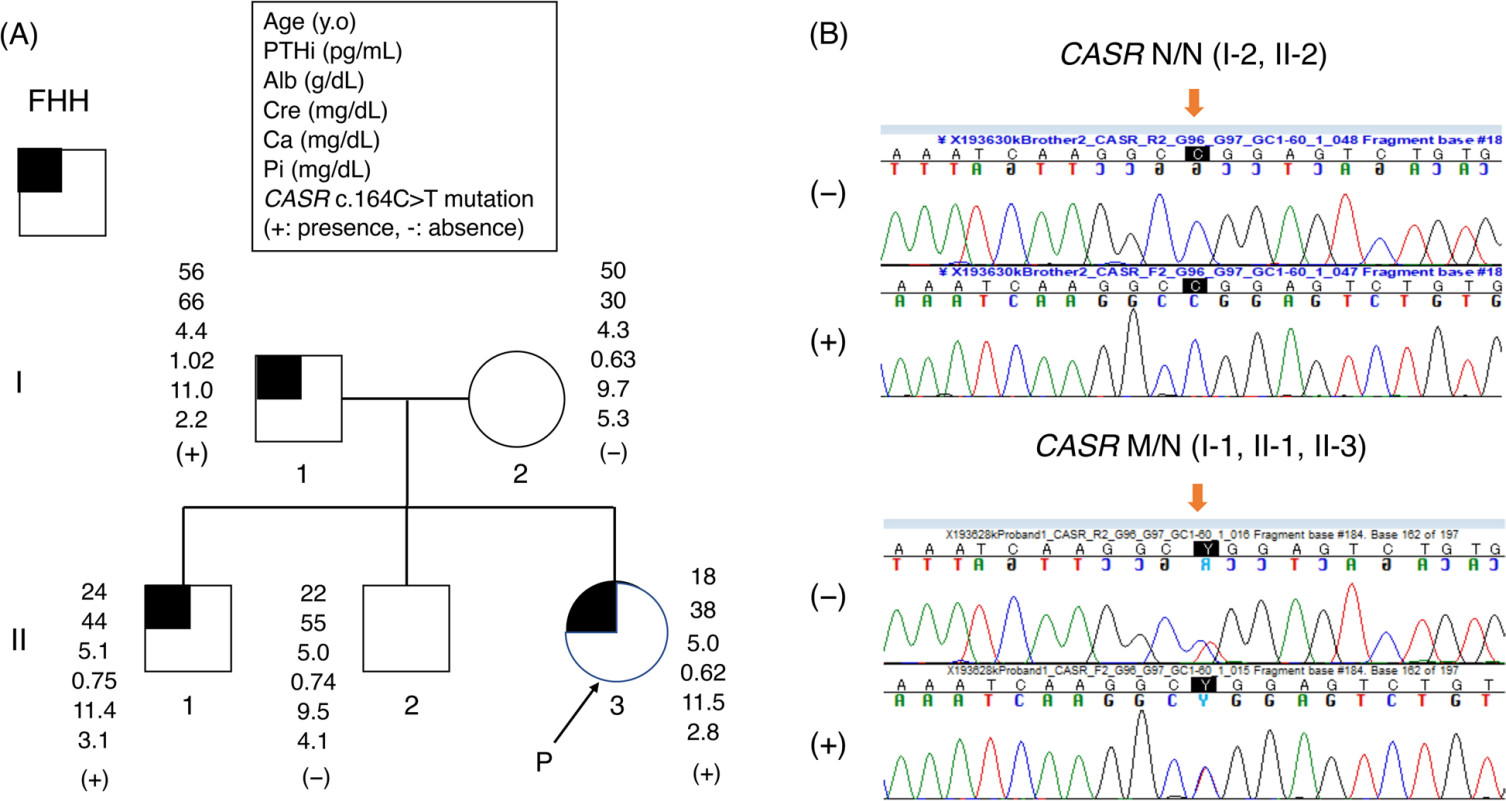
A family tree of the patient and the results of genetic testing in the patient and her family. **A** Squares, circles, and arrows indicate males, females, and proband, respectively. Family members, including I-1, II-1, II-3, had a history of hypercalcemia. d. Diseased. M and N, CASR wild type and p. Pro55Leu mutation alleles, respectively. Roman numerals on the left of the pedigrees indicate generation number, and the numbers below the symbols indicate the subject's number within each pedigree. The arrow shows the proband. **B** Sequence analysis of this family. M and N, CASR wild type and p. Pro55Leu mutation alleles, respectively. The arrow shows this mutation site (c.164). The half-filled symbols indicate the individuals with FHH

Since her brother (II-1) and father (I-1) had hypercalcemia (Fig. 2A), we proposed that she receive a genetic test to diagnose with FHH and she and her family agreed with our proposal. Whole exon sequencing was performed by fragmentation of DNA, enrichment of exonic regions using capture-based hybridization, PCR amplification, and rapid sequencing using a next-generation sequencer in Kazusa DNA laboratory. Exon sequencing of hereditary hypercalcemia-related genes (e.g., *MEN1, CDKN1B, RET, CASR, GNA11, AP2S1, CDC73, GCM2*) was performed and a pathogenic CASR mutation (CASR: NM_000388.4: exon2: c.164C > T: p. Pro55Leu) was noted in her father (I-1), brother (II-1), and the proband (II-3) (Fig. 2B), but no mutation in *GNA11* and *AP2S1* was identified. Based on the American College of Medical Genetics and Genomics Standards and Guidelines, the present variant was classified as pathogenic due to PS1 + PS3 + PM1 + PM2 [7]. She was then positively diagnosed as FHH1. As she was diagnosed with FHH, we followed up her with no medication. She and her family were relieved to hear that she needed no surgical treatment. Her plasma Ca^2+^ and Pi levels were almost unchanged for 18 months (Fig. 1).

## Discussion and conclusions

We report here a case of hypercalcemia with family history and low CCCR with hypovitaminosis D, in which genetic testing permitted definitive diagnosis of FHH1.

FHH is a very rare benign inherited condition that typically does not require parathyroidectomy. Patients with FHH usually have no symptoms and are often diagnosed by chance during routine blood examination. Weakness, fatigue, issues with concentration, constipation, polyuria, headache, and polydipsia have been reported by some people with FHH. The calcimimetic drugs are sometimes effective to improve hypercalcemic symptoms such as muscle aches, anorexia, polydipsia and constipation [2]. Rarely, people with this disorder experience pancreatitis or a chondrocalcinosis [8]. Although she had no past-history of pancreatitis and bone fractures, we may follow up her risk of pancreatitis due to hypercalcemia through her life [8].

In the current study, a heterozygous *CASR* mutation p.Pro55Leu was found to be pathogenic in the patient’s father (I-1), brother (II-1) and the proband (II-3). This mutation has previously been found in several individuals with FHH including one Japanese [2, 8–16]; it was classified as pathogenic in the ClinVar database (Variation ID: 279,731). The mutation is localized in the N-terminal extracellular domain (ECD) of CaSR, where ligand binding occurs; it causes a rightward shift of the PTH dose–response curve in response to extracellular calcium [11, 16]. Consistent with available information regarding the mutation, the patient showed relatively high PTHi despite the high plasma calcium level (Table 1 and Fig. 1).

In conclusion, we report a case of FHH1 due to *CASR* p.Pro55Leu mutation, in which only genetic testing allowed definitive diagnosis of FHH1 and thus avoided unnecessary surgery. The patient and family commented that they were very relieved to have found the cause of the disease. To diagnose with FHH by a genetic test is important to avoid unnecessary surgical treatment.

### Availability of data and materials

Clinical data from the corresponding author will be available upon request.

## Data Availability

The datasets used and analyzed during the current study are available from the corresponding author upon reasonable request.

## Abbreviations

FHH: Familial hypocalciuric hypercalcemia
PHPT: Primary hyperparathyroidism
CCCR: Calcium creatinine clearance ratio
CASR: Calcium sensing receptor
